# Enhanced Nogo-P3 amplitudes of mothers compared with non-mother women during an emotional Go/Nogo task

**DOI:** 10.1186/s40101-018-0167-9

**Published:** 2018-04-02

**Authors:** Sayuri Hayashi, Hiroko Wada, Sung-Phil Kim, Yuki Motomura, Shigekazu Higuchi, Yeon-Kyu Kim

**Affiliations:** 1 Department of Kansei Science, Graduate School of Integrated Frontier Sciences, Kyushu University, 6-10-1 Hakozaki, Higashi-ku, Fukuoka, 812-8581 Japan; 2 Department of Human Factors Engineering, Ulsan National Institute of Science and Technology, 50 UNIST-gil, Eonyang-eup, Ulju-gun, Ulsan, Republic of Korea; 3 Department of Human Science, Faculty of Design, Kyushu University, 4-9-1 Shiobaru, Minamiku, Fukuoka, 815-8540 Japan

**Keywords:** Emotion regulation, Mothers, Parental stress, Behavioral inhibition, Event-related potentials (ERPs), Nogo-P3

## Abstract

**Background:**

It is known that emotion regulatory responses of humans are changed by the experiences they have, but in particular, they are changed by becoming a mother. A recent study has found how a woman’s emotion regulatory response to a child’s crying changes after becoming a mother. However, mothers’ emotion regulatory responses other than those to children and the association between emotion regulatory response and parental stress are still unknown.

**Methods:**

Eighteen healthy Japanese females (nine mothers and nine non-mothers) participated in the experiment. They performed an emotional Go/Nogo task, with facial expressions of others (angry, happy, and neutral faces) used as emotional stimuli. The percentage of correct responses, response time, and event-related potentials (ERPs) during the task was measured.

**Results:**

This comparison revealed that the mother group had a larger P3 (Nogo-P3) amplitude than the non-mother group when Nogo trials were held. This indicates that in mothers, there was greater activation of the behavioral inhibition-related brain areas than in non-mother women when they inhibited inappropriate behavior following recognition of facial expressions of others. In addition, in the mother group, there was a negative correlation between parental stress levels and Nogo-P3 amplitudes evoked by angry faces. This suggests that there is a relation between the level of parental stress of mothers and their emotion regulatory responses to angry faces.

**Conclusions:**

Our results demonstrate that mothers’ emotion regulatory processes may differ from those of non-mothers in response, not only to a child’s crying but also to expressions of emotions by others, and also suggest that the inhibitory recognition activity of mothers can be affected by parental stress.

## Background

Emotion regulation, which is defined as the processes to control one’s experience and expression of emotion, is essential for humans to lead a social life. Recent studies suggest that it can be specifically changed by becoming a mother. These studies also describe the importance of emotion regulation to inhibit over-reactivity to children’s crying, which would be related to mental health of mothers [1–3].

Mentally and physically healthy mothers and non-mother women have different patterns of brain activity when they recognize negative emotion of children. Such a difference between mothers and non-mother women is observed in brain regions including the amygdala, insular cortex, anterior cingulate cortex, and ventral prefrontal cortex, and these areas related to regulating cognitive and emotional processes [4]. Other studies describe that the brain activities of mothers including brain regions related to emotion regulation are enhanced by negative emotion of own children [5, 6]. The abovementioned change in patterns of brain activity is considered to reflect the change in emotion regulation that results from becoming a mother. It has been also reported that the activity of the right lateral frontal pole and right inferior frontal gyrus in the prefrontal cortex is greater in mothers who display a high level of sensitivity to children than in mothers with a low sensitivity when they hear children crying [7]. In addition, it is suggested that the prefrontal cortex activation, especially in the left side [8], reflects ability to regulate an initial negative response to infant cries, making possible a more sensitive response to the infant [2]. Since these regions are associated with emotion regulation [9, 10], integration of information, and judgments to realize a goal [11], the level of sensitivity of mothers of infants is considered to be associated with activation of the brain function that affects the mothers’ emotion regulation, especially behavioral inhibition, when they see their children crying. It is considered that such characteristics of emotion regulation-related brain function of mothers are formed because emotion regulation (and the associated behavior) required for the period when women are the mother of an infant is different from that required for other periods of life [1, 2].

However, little is known about the brain activity associated with emotion regulation of mothers when they observe general emotional stimuli (other persons’ facial expressions, scenic images, etc.) other than a child’s cry. Given the fact that emotion regulation plays a crucial role in enabling humans to lead a social life, emotion regulation of mothers in situations often encountered in a general social life needs to be investigated. In addition, a correlation between emotion regulation and the level of parental stress, which is suggested to be associated with decreased sensitivity to children and inappropriate responses to infant behavior (e.g., abuse) [3], has not been clarified yet. In this study, therefore, we investigated emotion regulation of mothers when they recognized other persons’ facial expressions. Since previous studies have consistently found brain activity related to behavioral inhibition during emotion regulation, this study focused on such behavioral inhibition-related brain activity, particularly at the time of recognition of emotional stimuli. We also investigated a correlation between the level of parental stress and such brain activity.

For the evaluation of behavioral inhibition-related brain activity elicited by the recognition of emotional stimuli, we measured event-related potentials (ERPs) when subjects performed a Go/Nogo task using emotional stimuli. During a Go/Nogo task, Go trials that require subjects to execute a response and Nogo trials requiring them to withhold a response are presented. It is known that Nogo trials evoke two ERP components reflecting behavioral inhibition-related brain processing where each component reflects a different processing stage. The first component is called Nogo-N2, which is a negative component generated mainly in the frontal region about 200–400 ms after stimulus presentation. Nogo-N2 reportedly reflects one’s monitoring of competing choices and struggling to decide between the choices [12, 13]. The second component is called Nogo-P3, which is a positive component generated in the fronto-central region about 400–600 ms after stimulus presentation. Nogo-P3 is known to reflect brain processing associated with cognitive and behavioral inhibition [14]. When emotional stimuli are used as the stimulus presented in the Go/Nogo task, the influence of subjects’ emotion regulation is reflected in these ERP components. For example, it has been reported that highly impulsive individuals had larger Nogo-P3 amplitudes than less-impulsive individuals only when stimuli with a high emotional valence were presented as the Nogo stimulus [15]. Furthermore, in this study, we also examined a correlation between parental stress of mothers and their ERPs when performing the Go/Nogo task, to investigate the influence of parental stress on emotion regulation. Parental stress of mothers can be measured with a questionnaire called the Parental Stress Index (PSI) form, and the total PSI score obtained from the questionnaire can indicate the level of overall parental stress of mothers of infants [16].

Based on the considerations above, we draw the following two hypotheses to be addressed:

a) If mothers of infants have more activity in brain areas related to emotion regulation, especially behavioral inhibition, even when general emotional stimuli are presented, the Nogo-2 and Nogo-P3 of mothers should have larger amplitudes than non-mother women. Based on previous studies [2, 8], we expected that such characteristics of mothers would be more clearly observed in the left hemisphere.

b) If the level of parental stress of mothers of infants correlates with their behavioral inhibition-related brain activity following recognition of emotions, there should be a correlation between the total PSI score of mothers and their Nogo-N2 and Nogo-P3 amplitudes.

## Methods

### Participants

Twenty-four healthy Japanese females participated in the experiment. Of these, 13 females were engaged in raising their biological child who was 3 years old or under (the mother group) and 11 females had never experienced pregnancy, child birth, or child rearing (the non-mother group). The ages of the mother group and non-mother group were 35.9 ± 2.29 (mean ± SD) years old and 27.2 ± 2.89 years old, respectively. Responses to a questionnaire indicated that the mother group had normal affection for their biological child. The participants received an adequate verbal/written explanation of the experiment, agreed to participate in the experiment, and signed a written consent form before participating in the measurement. This experiment was carried out with approval of the Ethics Committee of Kyushu University.

### Stimuli and task

**Fig. 1:**
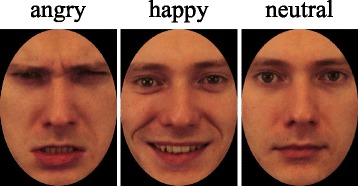
Example of the stimuli used in the emotional Go/Nogo task. These pictures are the example of the facial stimuli used in this study. The task included images of angry, happy, and neutral faces

Pictures of facial expressions from 10 adults (five males and five females) selected from the Karolinska Directed Emotional Faces (KDEF) set [17] were used as the stimuli presented in the task. In consideration of a preceding study [18], pictures of these 10 actors (angry/happy/neutral faces) were selected as stimuli. The pictures of these faces were cropped to an elliptical shape to eliminate the influence of hair and background cues (Fig. 1). Each face subtended a visual angle of approximately 4° × 3°. The task comprised of six blocks, with each block consisting of two categories of facial expression pictures: angry-happy/angry-neutral/happy-angry/happy-neutral/neutral-angry/neutral-happy. In each stimulus pair, one was used as a Go stimulus and the other was used as a Nogo stimulus. For example, in the pair of “angry-happy”, angry is used as a Go stimulus and happy as a Nogo stimulus in one block. The participants were given instructions to press a button as soon as possible with the index finger of their right hand which was their dominant hand when a Go stimulus was presented and to withhold a response when a Nogo stimulus was presented. Just before starting each block, the participants were informed which emotional expression was a Go stimulus and which was a Nogo stimulus. Each block contained 120 trials, of which 90 trials (75%) were Go and 30 trials (25%) were Nogo. The order of the blocks and the order of the trials were randomized between the subjects. Each stimulus was presented for 500 ms, with an inter-trial interval of 1750 ± 250 ms. Presentation Ver. 18.1 (Neurobehavioral Systems, Inc., USA) was used to present stimuli during the task.

### Questionnaire

As a questionnaire form to measure parental stress, the Japanese version of the Parental Stress Index (PSI) form [16] was used. The PSI is an academic tool developed by Abidin in 1980s to measure parental stress of mothers, and the Japanese version of the PSI form was redeveloped to modify the original PSI suitable for Japanese parents. It contains 78 items, consisting of 38 items and 7 subscales to measure the stress related to characteristics of children (Child Domain) and 40 items and 8 subscales to measure the stress related to parents themselves (Parent Domain). The total PSI score summing the scores from all the items was used as an index of the overall parental stress of the mother group.

### Procedure

The experiment was carried out in a sound attenuating and electromagnetic wave shielded artificial climate room. First, the participants were seated in a chair in the experiment room and received an explanation of the experiment. Next, they performed practice trials to become familiar with the task. Then, the task and concurrent electroencephalogram (EEG) measurement were carried out. During the task, adequate breaks were taken between trials if needed. After the task, the mother group participants answered the PSI questionnaire.

### Recording and analysis of data

Task response times and answers were recorded using Presentation Ver. 18.1 (Neurobehavioral Systems, Inc.). Response times and percentage of correct responses were computed from the recorded data. Response time was defined as the time elapsed from Go stimulus presentation to pressing the button, and percentage of correct responses was defined as the number of successful trials divided by the number of total trials under each facial expression (angry, happy, neutral). Trials were regarded as successful if the button was pressed 100 to 1000 ms after a Go stimulus presentation, and if the button was not pressed between 0 and 1000 ms after a Nogo stimulus presentation. Other trials were regarded as errors and excluded from the analysis.

EEG was recorded with a 64-channel EEG cap (64-channel HydroCel GSN; Electrical Geodesics Inc., USA). The EEG signals were amplified by a biological amplifier (Net Amps 200 64-channel EEG Amplifier; Electrical Geodesics Inc.) and were recorded using specialized software (Net Station, ver. 4.1.2; Electrical Geodesics Inc.). During the measurement, EEG was continuously recorded at a sampling frequency of 250 Hz with the impedance of electrodes kept at 100 kΩ or less. The low-cut and high-cut frequencies of the hardware filter were set to 0.1 Hz to 100 Hz, respectively. Although a lower cutoff frequency (e.g., 0.01 Hz) is usually recommended for ERP experiments, the current setting was acceptable and often used with a high-impedance recording system like the one we used. A reference electrode was located at Cz in the International 10-20 system.

EEG interpretation software (EMSE Data Editor 5.5.2; Source Signal Imaging Inc., USA) was used for the analysis of the measured EEG data. In the preprocessing stage, EEG signals from electrodes near the ears and cheeks containing artifacts were excluded from the analysis. The average potential of the remaining 58 electrodes was used as a reference for the analysis. In addition, EEG was filtered again offline with an IIR filter (12 dB/octave), with low-cut frequency of 0.5 Hz and a high-cut frequency of 30 Hz.

For the computation of ERPs, the epoch between 200 ms before and 800 ms after stimulus presentation was set apart as a single trial. All of the individual trials under one condition were averaged to obtain an ERP waveform, excluding the ones with incorrect responses or with noise visually inspected in the period between − 500 ms and 1000 ms after stimulus onset (such as an eye blink and muscle artifacts, EEG amplitudes outside the range of ± 60 μV). Before calculating the mean of all the trials, waveforms were aligned to the average potential over the 200-ms pre-stimulus epoch of each trial as the baseline. The arithmetic mean number of trials used under each condition per member of the mother group was Angry-Nogo, 39.9, Happy-Nogo, 43.1, and Neutral-Nogo, 42.7. Those of the non-mother group were Angry-Nogo, 43.0, Happy-Nogo, 39.4, and Neutral-Nogo, 43.4.

**Fig. 2:**
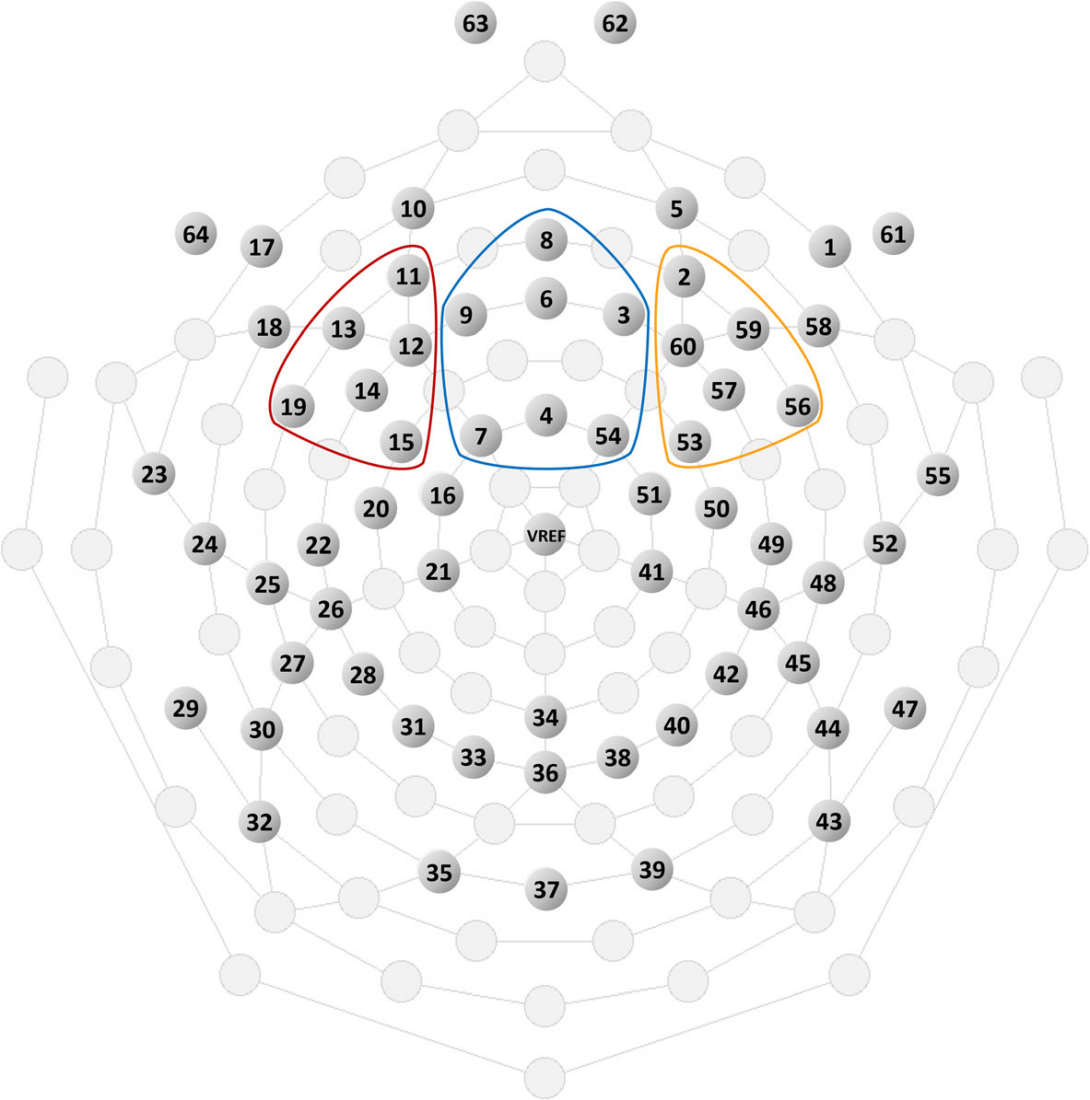
Electrode positions and three areas of interest. The blue, red, and orange solid-line circles indicate three areas of interest at the fronto-central region (FCz, FC3, and FC4, respectively). FCz, FC3, and FC4 mean the areas of interest around FCz, FC3, and FC4 according to the 10-20 system

**Fig. 3:**
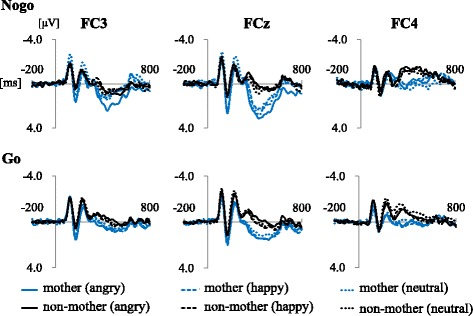
Average ERP waveforms and N2 and P3 components in each area of interest. The black line and the blue line indicate waveforms of the mother group and non-mother group respectively. The solid line, dashed line, and a dashed-dotted line indicate waveforms elicited by angry, happy, and neutral faces respectively. The peak amplitude between 200 and 300 ms and the average amplitude in the epoch between 400 and 600 ms were defined as N2 component and P3 component respectively

Global field power (GFP) [19] was derived from the computed ERPs. Based on GFP and preceding studies, N2 and P3 were defined as the peak potential between 200 and 300 ms after stimulus presentation and the average potential between 400 and 600 ms after stimulus presentation, respectively. Furthermore, three areas located at the fronto-central region (FCz 3, 4, 6, 7, 9; 54, FC_3_ 11, 12, 13, 14, 15, 19; FC_4_ 2, 53, 56, 57, 59, 60) were set as areas of interest, and N2 and P3 amplitudes in these three areas were computed (Figs. 2 and 3).

The PSI questionnaire was administered and scored in accordance with the methods explained in the Manual for the Japanese version of Parental Stress Index, and then the total PSI score was calculated.

### Statistical analysis

When computing ERPs, it was found that the total number of trials under certain condition was less than 20 for some participants (four participants from the mother group and two participants from the non-mother group). The data of these participants was excluded from the statistical analysis, leaving the data of nine participants from the mother group (36.0 years old ±1.93) and the data of nine participants from the non-mother group (26.6 years old ±2.82) for the subsequent statistical analysis. There was a statistical difference in the age of the two groups (*p* < 0.001). All of them were right-handed and had normal or corrected to normal vison. The age of the children of the mothers was 25.5 ± 13.9 months (mean ± SD). All of the mother group were married and lived with their husbands and children. There was no participant who was feeding her child only through breast-feeding.

A three-way analysis of variance (ANOVA) was performed with group (mothers/non-mothers), emotion (angry/happy/neutral), and brain area (FC3/FCz/FC4) as factors influencing Nogo-N2 and Nogo-P3 amplitudes. A two-way ANOVA also was performed with group and emotion as factors influencing response time. Degrees of freedom were corrected with the Greenhouse-Geisser method. The Bonferroni-Holm method was used to adjust the *P* value during post hoc tests. In addition, Pearson’s correlation coefficients were calculated for the PSI and ERP components under each condition. Non-correlation tests were performed upon the calculated correlation coefficients to confirm the significance of the correlation. All of the significance levels for these tests were set to 5%.

## Results

### Behavioral data

#### Percentage of correct responses

The mean percentages of correct responses of the mother group and of the non-mother group were 0.95 ± 0.07 (mean value±SD) and 0.94 ± 0.11, respectively (Table 1). A three-way ANOVA was performed on group, trial, and emotion as factors affecting the percentages of correct responses, which revealed a main effect of trial (*F*(1,16) = 34.9, *p* < 0.001). Emotion had a main effect (*F*(2,32) = 10.5, *p* = 0.001), and the percentages of correct responses to angry faces were lower than those to other faces (angry vs neutral: *t*(16) = 3.80, *adj.p* = 0.001, angry vs happy: *t*(16) = 3.40, *adj.p* = 0.007). There were no significant main or interaction effects of the group.

**Table 1:** The means and SDs of percentage of correct responses

Percentage of correct responses (%)	Nogo trials			Go trials		
	Angry	Happy	Neutral	Angry	Happy	Neutral
The mother group	87.0 (8.7)	88.9 (6.0)	91.2 (7.5)	93.5 (3.5)	99.5 (0.6)	98.1 (2.4)
The non-mother group	79.1 (12.1)	82.3 (8.3)	82.5 (9.4)	96.5 (5.0)	98.8 (1.7)	97.8 (3.3)

*Notes:* Data are mean (SD)

### Response time

With respect to the response time to correct Go trials, a two-way ANOVA was performed on group and emotion as factors, which revealed a significant main effect of emotion (*F*(2,32) = 3.87, *p* = 0.041) (Table 2). The multiple comparison test was performed for the further analysis of the main effect of emotion, but this revealed no significant result. There were no significant main or interaction effects of the group.

**Table 2:** The means and SDs of response times in correct Go trials

Response times (ms)	Angry	Happy	Neutral
The mother group	418.6 (53.1)	398.3 (23.7)	414.9 (31.8)
The non-mother group	442.5 (52.6)	421.1 (57.1)	428.6 (57.9)

*Notes:* Data are mean (SD)

### ERP data

#### Nogo-N2

**Fig. 4:**
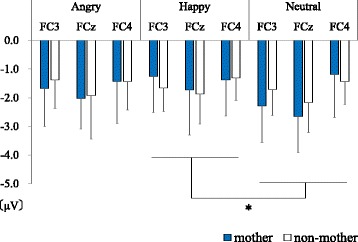
Nogo-N2 amplitudes in FC3. The gray column and the blue column indicate data of the mother group and the non-mother group respectively. The Nogo-N2 amplitudes evoked by happy faces were smaller than the Nogo-N2 amplitudes by neutral faces. *p < 0.05

A three-way ANOVA was performed with group (mothers/non-mothers), emotion (angry/happy/neutral), and area (FC3/FCz/FC4) as factors affecting the Nogo-N2 amplitudes (Fig. 4). Area was revealed to have a main effect (*F*(2,32) = 6.73, *p* = 0.005). A post hoc test revealed that the Nogo-N2 amplitudes in FCz were larger than the other two areas (FCz vs FC3: *t*(16) = 2.48, *adj.p* = 0.049, FCz vs FC4: *t*(16) = 3.25, *adj.p* = 0.015). There was no significant difference between FC3 and FC4 (*t*(16) = 1.54, *adj.p* = 0.143). Emotion had a main effect (*F*(2,32) = 4.84, *p* = 0.020), and the Nogo-N2 amplitudes evoked by happy faces were smaller than the Nogo-N2 amplitudes by neutral faces (*t*(16) = 3.90, *adj.p* = 0.004). There were no significant main or interaction effects of the group.

#### Nogo-P3

**Fig. 5:**
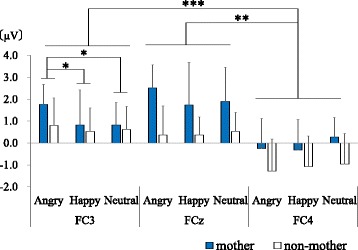
Nogo-P3 amplitudes in FCz. The gray column and the blue column indicate data of the mother group and the non-mother group respectively. The Nogo-P3 of the mother group were larger than those of the non-mother group. Only in the FC3, the Nogo-P3 evoked by angry faces were larger than those by other two emotions. *p < 0.05, **p < 0.01, ***p < 0.001

A three-way ANOVA was performed with group (mothers/non-mothers), emotion (angry/happy/neutral) and area (FC3/FCz/FC4) as factors affecting the Nogo-P3 amplitudes (Fig. 5). Group was observed to have a main effect (*F*(1,16) = 8.45, *p* = 0.010), with the Nogo-P3 amplitudes of the mother group being larger than those of the non-mother group. Area also had a main effect (*F*(2,32) = 17.32, *p* < 0.001). A post hoc test was performed, which revealed the Nogo-P3 amplitudes in FCz and FC3 were larger than FC4 (FCz vs FC4: *t*(16) = 6.06, *adj.p* < 0.001, FC3 vs FC4: *t*(16) = 3.60, *adj.p* = 0.005). There was no significant difference between FCz and FC3 (*t*(16) = 1.32, *adj.p* = 0.205). A marginally significant interaction effect between emotion and group was observed (*F*(2.32) = 2.51, *p* = 0.074). Post hoc tests showed that the Nogo-P3 amplitudes evoked by angry faces were larger than the Nogo-P3 amplitudes evoked by the other two emotions only in FC3 (Angry vs Neutral: *t*(16) = 2.68, *adj.p* = 0.049, Angry vs Happy: *t*(16) = 2.63, *adj.p* = 0.049). There was no significant difference between the Nogo-P3 amplitudes elicited by happy and neutral faces (*t*(16) = 0.30, *adj.p* = 0.769). Other interaction effects were not significant.

We additionally performed a three-way ANOVA with group, trial, and emotion as factors affecting the Go-P3 amplitudes. Area was observed to have a main effect (*F*(2.32) = 5.45, *p* = 0.014), with the Go-P3 amplitudes in FCz which were larger than FC4 (*t*(16) = 3.16, *adj.p* < 0.018). Group had a marginally significant main effect; however, it was not significant (*F*(1.16) = 4.00, *p* = 0.063). Other main or interaction effects were not significant.

### Correlation between parental stress and ERP components

**Fig. 6:**
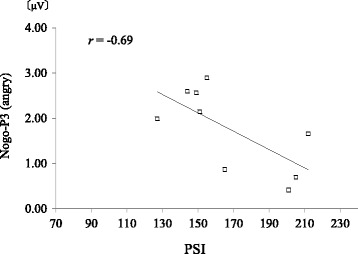
Correlation between PSI and Nogo-P3 amplitude in FC3 evoked by angry faces. □ indicates the data of each participant in the mother group. The vertical axis and the horizontal axis represent the Nogo-P3 amplitude evoked by angry faces in FC3 and the total PSI score respectively. There was a negative correlation between the mother group’s total PSI score and the Nogo-P3 amplitude in FC3 evoked by angry faces

The average total PSI score of the mother group was 167.7 ± 30.6, and all participants reported a score within the normal range. Pearson’s correlation coefficients were computed and non-correlation tests were performed regarding the total PSI score and ERP components in each area under each condition. The results revealed a significant negative correlation between the total PSI score and the Nogo-P3 amplitude in FC_3_ evoked by angry faces (*r*^2^ = 0.47, *p* = 0.042) (Fig. 6). There was no significant correlation between the total PSI score and other ERP components.

## Discussion

With regard to the first hypothesis, a difference between mothers and non-mothers was clearly shown in the larger Nogo-P3 amplitude of the mother group (Fig. 5). This result suggests that there is a difference between the processing of mothers and non-mother women that is reflected in the Nogo-P3 amplitude. Considering the result that the Nogo-P3 amplitudes elicited by angry faces were larger than the other faces only in FC3, the Nogo-P3 amplitude would seem to reflect not only brain activities inhibiting recognition and behavior following presentation of emotional stimuli [13], but also processes related to emotion. This idea is in line with a previous study which suggests that Nogo-P3 amplitudes overlap with the automatic response inhibition of emotions during implicit emotional tasks [20]. This interpretation does not contradict previous neuroimaging studies of emotion regulation and maternal brain activity [2, 8]. However, we should be careful because the present study did not perform source analysis and because there are few previous studies about left-right differences in ERP during emotional Go/Nogo tasks.

Based on previous studies focused on maternal brain activities, especially emotion regulation of mothers [1, 2], we expected that the differences between mothers and non-mothers would be enhanced in negative emotional conditions. However, the results of this study indicate that the Nogo-P3 amplitudes of mothers were larger than those of non-mothers, regardless of the emotion of the facial stimuli. Thus, when inhibiting impulsive behavior, mothers may tend to exhibit greater brain activity related to inhibition. It is reported that impulse control disability in mothers is related to child maltreatment [21], and it has been suggested that stress during childhood including child maltreatment potentially leads to long-term changes in learning, behavior, and physiology that result in higher levels of stress-related chronic diseases or the prevalence of unhealthy lifestyles [22]. The importance of impulse control ability in mothers may have a connection with the enhanced Nogo-P3 amplitudes in mothers observed here.

With regard to the second hypothesis, association between the mother group’s parental stress and inhibitory brain activity when emotions of others are recognized was supported by the negative correlation between the total PSI score and the Nogo-P3 amplitude when angry faces were presented as the Nogo stimulus (Fig. 6). This indicates that there may be an association between a high level of overall parental stress and a low level of brain activity inhibiting recognition and behavior in response to angry faces. These inhibitory brain activities may be associated with inhibitory control and emotional intelligence, which are important for human social behavior [23]. Considering these, parental stress in mothers of infants may be associated also with social behavior of mothers. This point would be possible to extend to mothers displaying an abnormal parental stress score as well, and need further investigation in the future.

There are some limitations to the reliability of this study. First, the lifestyle (for example, living together or alone and sleeping habits), age, and menstrual cycle in the two groups were not controlled. It was too difficult to control these because these are related to characteristics of mothers, who for example take care of their children day and night. However, these factors should be controlled in subsequent studies if possible. Second, the number of participants may not have been sufficient. It is almost the same as some previous studies which investigated the differences between mothers and non-mother women, for example, Proverbio et al. [24], but it is advisable to use more participants [25]. This study has weaknesses regarding group selection and sample size, but it may be the first study indicating that mothers exhibit greater brain activity related to inhibition when inhibiting impulsive behavior in response to emotional and non-emotional stimuli than non-mother women. This study should be useful as a preliminary study in the area of emotion regulation by mothers.

## Conclusions

The results of this study revealed that mothers and non-mother women have differences in their emotion regulation, especially in brain activity associated with behavioral inhibition. The difference was clearly observed in Nogo-P3 amplitudes during the Go/Nogo task with emotional stimuli. This indicates that compared to non-mother women, mothers have greater brain activity inhibiting recognition and behavior while performing appropriate behavior (inhibiting inappropriate behavior) in response to facial expressions of others. In addition, there was an association between a high level of parental stress and a low level of behavior inhibition-related brain activity in response to angry faces, suggesting that parental stress of mothers of infants may be associated with social behavior of mothers.

## Abbreviations

ANOVA: Analysis of variance
EEG: Electroencephalogram
ERPs: Event-related potentials
GFP: Global field power
KDEF: The Karolinska Directed Emotional Faces
PSI: The Parental Stress Index
